# A Georeferenced Dataset for Mapping and Assessing Subgrade Defects in China’s High-Speed Railways

**DOI:** 10.1038/s41597-024-03112-7

**Published:** 2024-03-14

**Authors:** Jinchen Wang, Luqi Wang, Yinsheng Zhang, Jingyu Zhang, Jianlin Li, Sen Li

**Affiliations:** 1 Key Laboratory of Geological Hazards on Three Gorges Reservoir Area of Ministry of Education, China Three Gorges University, Yichang, 443002 Hubei P. R. China; 2 School of Environmental Science and Engineering, Huazhong University of Science and Technology, Wuhan, 430074 Hubei P. R. China; 3 School of Artificial Intelligence and Automation, Huazhong University of Science and Technology, Wuhan, 430074 Hubei P. R. China; 4 College of Hydraulic and Environmental Engineering, China Three Gorges University, Yichang, 443002 Hubei China

**Keywords:** Civil engineering, Environmental impact

## Abstract

China has the world’s longest high-speed rail (HSR) network, marked by dense transportation and complex operations. However, frequent train use coupled with extreme weather conditions has led to rising subgrade issues. Existing railway defect records suffer from inconsistency, hindering direct applicability. Currently, there is a lack of a relevant dataset dedicated to HSR subgrade defects. To bridge this gap, we developed a comprehensive georeferenced dataset that encompasses defect records extracted from peer-reviewed literature published between 1999 and 2023 in China. Rigorous quality control procedures were implemented to eliminate duplicate data and ensure the accuracy of the dataset. The dataset consists of georeferenced records for eight different defects, spanning across 661 locations and categorized at various scales, ranging from provinces to townships. The most commonly reported defect types include subgrade settlement, frost damage, uplift deformation, and mud pumping. This dataset provides a comprehensive map of historical subgrade defects affecting high-speed railways in China. It could facilitate operational risk assessments and the prediction of subgrade performance.

## Background & Summary

Since the opening of the world’s first high-speed railway (HSR), the Japanese Shinkansen, in 1964, high-speed rail systems have been widely used within the realm of transportation^1^. Due to their elevated operational speed, high passenger density, and large capacity, alongside geological heterogeneities tracing the railway’s trajectory, a spectrum of defects inevitably arises in the subgrade under long-term train loads^2,3^. These defects have caused train speed restrictions, delays, and even serious safety accidents^4^, which have consistently attracted attention and concern. Furthermore, these defects exhibit a seasonal occurrence pattern concerning time and demonstrate a cumulative, recurrent character concerning space. Under the influence of changing climatic and environmental conditions, the timely detection of new subgrade defects might be impeded^5^.Therefore, there is an urgent need to better understand the distribution of subgrade defects that pose potential safety threats, thereby facilitating an enhanced evaluation of the operational vulnerabilities of high-speed rail^6–8^.

China has the world’s most extensive operational HSR network, the largest scale of projects under construction, and the highest number of operating trainsets^9^. Several recent studies have reviewed and compiled the occurrence records of track subgrade defects within China’s HSR, focusing on rail routes such as the Harbin-Dalian^10^, Lanzhou-Xinjiang^11^, and Shanghai-Nanjing lines^2^. However, a comprehensive and systematic description of the geospatial distribution of subgrade defects within the expanse of Chinese HSR infrastructure remains notably absent^12–14^. Additionally, given that existing subgrade defect datasets either lack spatial information or exhibit limited spatial resolution, the availability of a dataset offering more detailed geographic information at a fine scale would be beneficial in identifying risk areas and modeling environmental and safety management practices^15,16^.

This dataset encompasses 661 georeferenced subgrade defect occurrence records reported from 1999 to 2023. It covers eight types of defects reported across 239 districts and counties in China. The most frequently reported subgrade defects include subgrade settlement^17–19^, uplift deformation^20^, frost damage^21,22^, and mud pumping^2^.Moreover, the main causes of defects were recorded, with 73 and 54 defect records related to construction and design errors, respectively. These records constitute a valuable resource, facilitating the identification of regions susceptible to defects and formulation of targeted environmental and safety management strategies which underpin the reliable and secure operation of high-speed railways across China^23,24^.

## Methods

**Fig. 1:**
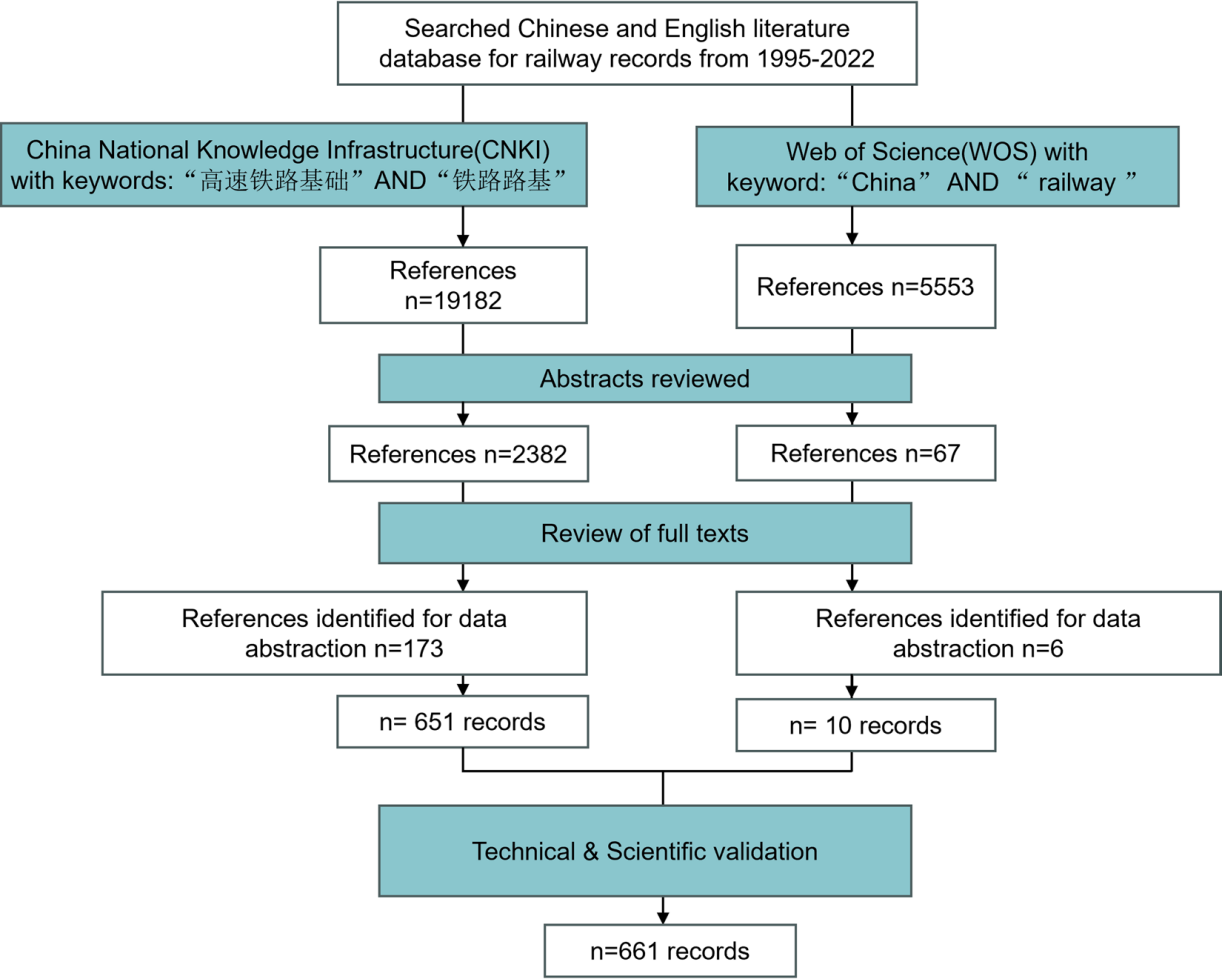
Schematic overview of the literature search procedure and results.

Our data collection procedures, illustrated in Fig. 1, involved an extensive literature review sourced from two prominent scientific citation indexing platforms: the Web of Science (WOS) (https://www.webofscience.com) and China National Knowledge Infrastructure (CNKI) (http://www.cnki.net/). We opted to exclude PubMed from our search due to its significant overlap with WOS and CNKI, particularly in terms of containing English abstracts of Chinese literature already available in CNKI. The literature search timeframe was set from 1999 to 2023, ensuring inclusion of all papers published up to April 2023, including those from 2022. We employed the terms (“China” AND “railway”) with WOS and (“高速铁路基础” AND “铁路路基”) with CNKI. Publications, including journal articles, conference proceedings, and degree theses, were retrieved if the specified terms appeared in any part of their content. No language restrictions were imposed on these searches.

A total of 24,735 articles were retrieved, with 19,182 from CNKI and 5,553 from WOS. Firstly, a literature review was conducted to eliminate duplications. Secondly, the abstracts of the literature were scrutinized, and publications solely focusing on railway economy and railway power supply, without relevance to railway construction, were excluded^25^. This process resulted in 2,449 papers for the subsequent full-text review, from which location information of defect occurrence was further extracted. Thirdly, after a thorough examination of all available full-text information, publications that did not report the location of the defects were excluded. Finally, 179 publications were confirmed to be eligible for data extraction. The earliest Chinese article, published in 1999, reported on the train shutdown caused by the genetic collapse of railway roads.

The literature review yielded several key pieces of information that were retained in the dataset, including (i) name of HSR line, (ii) the specific classification of HSR defects, (iii) the latitude and longitude coordinates of the occurrence record, (iv) the time when the defect was reported, and (v) the primary cause of each defect. To safeguard data fidelity and eliminate duplications, a comprehensive review of the dataset was conducted by a second evaluator. In many regions, such as Menyuan Hui Autonomous County and Runzhou District, the occurrences of defect have been observed in a persistent and extensive manner^2,10^. Consequently, a corresponding extension of the defect statistics timeframe has been implemented. It is essential to underscore that certain subgrade defects may not be recorded if they fail to meet the criteria outlined in China’s national standard “Code for Design of High-Speed Railways” (TB10621-2014)^26,27^. The final dataset comprises 661 records of HSR defects, consisting of 233 cases of subgrade settlement (settlement values ranging from 5 to 2300 mm), 88 cases of uplift deformation (ranging from 5 to 122 mm), 254 incidents of frost damage (frost heave values ranging from 4 to 50 mm), 29 cases of mud pumping, five cases of soil extrusion, 26 instances of slope failure, six cases of deterioration of protection structures, and 20 cases of poor drainage.

### Geo-positioning

To extract the location details from the literature, we initially addressed the conversion of the high-speed rail mileage information into latitude and longitude coordinates^28^. This conversion is essential since many publications solely indicate the occurrence of defects with reference to the mileage along HSR. Next, considering the potential presence of duplicated records detailing HSR foundation defects at the same location across different publications, it is anticipated that duplicate entries of records might arise. We thus aggregated location records to reduce repeated geo-positioning operations which could induce redundancy and error. A total of 239 unique locations were identified, and their geospatial coordinates (longitude and latitude) were determined using a range of geospatial tools. These tools include xGeocoding software (http://www.gpsspg.com/xgeocoding/), which incorporates APIs to access georeference functions of major online map services in China, namely, Baidu Map, Tencent’s QQ Map, and Amap. Additionally, Google Earth (http://www.google.co.uk/intl/en_uk/earth) was utilized when necessary, and simple keyword searches with Google or Baidu were employed as a last resort.

Following previous studies^15,16^, we further classified all the locations into four different levels according to their geographic scales and administrative levels, i.e., provincial, prefectural, county, and township and finer level. This systematic categorization aims to facilitate the efficient utilization of this dataset by enabling users to selectively extract pertinent segments. Subsequently, the occurrence locations of HSR defects were visualized on the administrative boundary map (2015) obtained from the CAS Resource and Environmental Data Cloud Platform (http://www.resdc.cn/data.aspx?DATAID=202).

## Data Records

In the China HSR subgrade defects distribution dataset (available from figshare^29^), each row represents a record of the occurrence of roadbed disease at a particular location reported in a specific year. The dataset columns are explained below:

1.rai_na: Name of railway line.
2.def_ty: Identifying the types of defects (definitions of defects are shown in Table 1).

**Table 1:** Definition of HSR Subgrade Defect.

Type of defect	Definition	Type of defect	Definition
Subgrade settlement	Settlement refers to the vertical deformation that occurs over a small or extensive area due to inadequate compaction of subgrade soil, insufficient depth of foundation treatment, damage between piles, creep of underlying soil layers, or regional settling.	Frost damage	In cold regions, subgrade and its protective structures experience uneven frost heave under low temperature conditions, leading to issues like tilting and cracking of protective structures.
Mud pumping	This defect occurs in areas with poor drainage. Repeated vibrations from train traffic cause softening or thixotropic liquefaction of the sub-ballast, leading to the formation of mud slurry.	Uplift deformation	This happens when expansive soils or rocks within the subgrade or its base react with external moisture, causing the subgrade to arch upwards.
Soil extrusion	This deformation occurs when sub-ballast soil undergoes shear failure under the load of the train, leading to lateral squeezing.	Slope failure	Slope slipping and collapsing refer to the shallow sliding or collapse of soil slopes.
Deterioration of protection structures	These defects in retaining structures occur due to natural environmental factors, uneven settling of foundations, and issues in construction and design.	Poor drainage	Poor or insufficient drainage leads to rainwater penetrating into or eroding the subgrade, deteriorating the quality of the subgrade or its stability.
3.lon: The longitudinal coordinate of the location of subgrade disease occurrence (WGS1984 Datum).
lat: the latitude of the location of subgrade disease occurrence (WGS1984 Datum).
loc_level: the geographic scale of location (1 = provincial level, 2 = prefectural level, 3 = county level, 4 = township or finer level).
loc_l1: provincial level information of the location (name of province, autonomous region, municipality, or special administrative region of China).
loc_l2: prefectural level information of the location (name of prefectural-level city, or autonomous prefecture).
8.loc_l3: county level information of the location (name of county-level city, autonomous banner, district or county)
loc_l4: township or finer level information of the location.
loc_ref: supplemental geographical information of the location.
11.def_ca: main cause of defect occurrence.
12.def_va: defect detection values (mm).
col_stt: start year of subgrade disease sampling/collection
col_end: end year of subgrade disease sampling/collection
pub_t: the year of the publication.
pub_id: identification number of references (those start with letters ‘c’ and ‘w’ indicate that the reference is retrieved from CNKI and WOS, respectively).
pub_full: references identified for data extraction.
pub_link: doi/links to references.

**Fig. 2:**
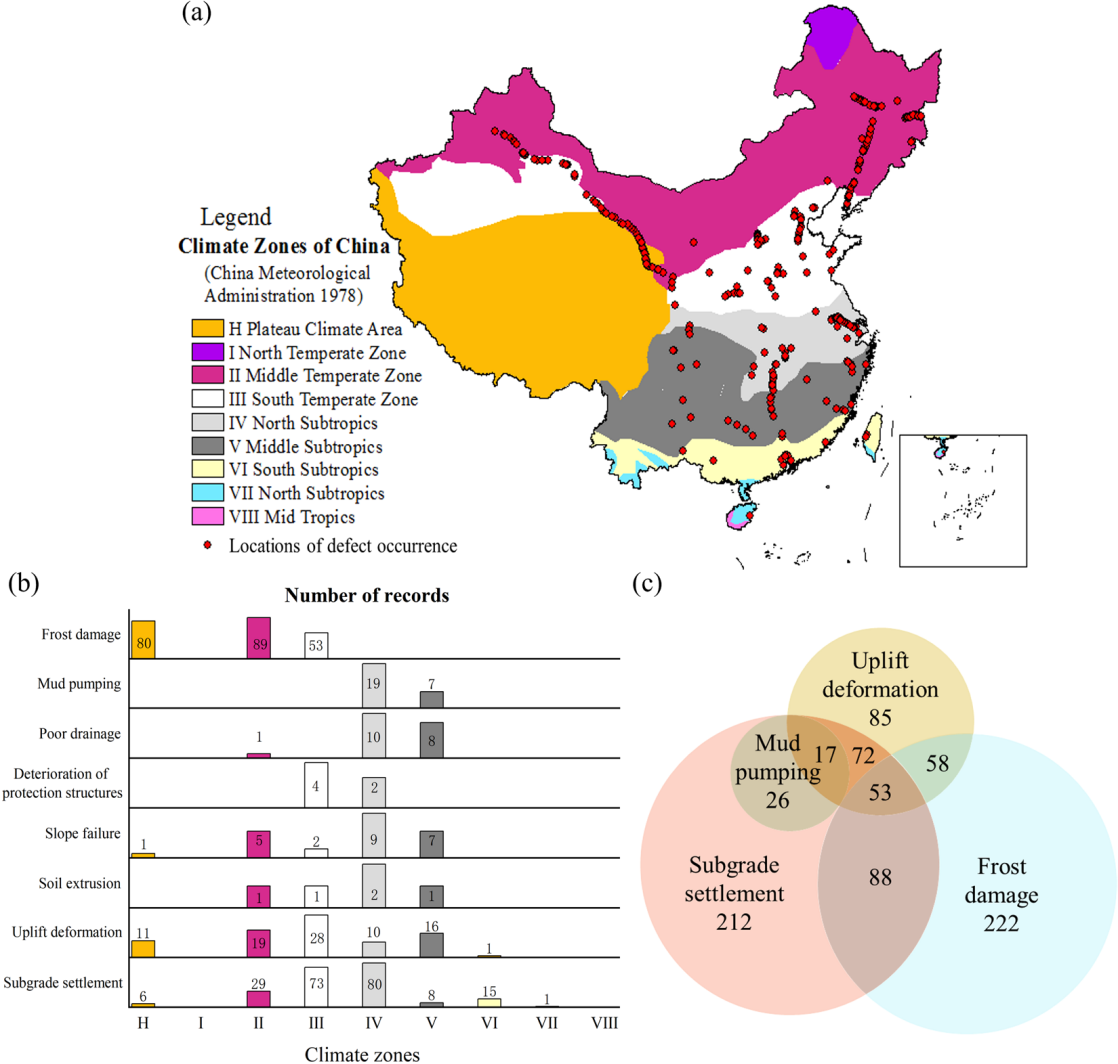
Distribution of subgrade defect occurrence records across different climate zones in China: (**a**) Geolocations of subgrade defect occurrences in various climate zones; (**b**) Quantity of subgrade defects in diverse climatic zones; and (**c**) Co-occurrence patterns of subgrade defect types within the same climate zones.

We obtained the distribution of subgrade defects in different climate zones from the data, as shown in Fig. 2. Investigates primarily focused on subgrade settlement, frost damage, uplift deformation, and mud pumping. The occurrence of these defects correlates closely with local climatic and geological conditions. For instance, frost damage is concentrated in the mid-latitude temperate zone, characterized by long cold winters and high humidity, leading to soil freezing and heaving^30,31^. Conversely, mud pumping defects are more common in the southeast, where heavy rainfall infiltrates the foundation bed, causing settlement^32^. Moreover, the subgrade’s uplift deformation is linked to the micro-expansion of the filler used. Within the same climate zone, multiple defects often coexist, thereby introducing complexity to the overall condition of subgrade. To illustrate, subgrade settlement, frost damage, and uplift deformation were reported in 53 locations within a single climate zone, accounting for 9.7% of the total defect occurrence locations. This complexity underscores the need for pre-emptive and remedial measures to address these multifaceted challenges^33–35^.

## Technical Validation

A total of 661 records documenting defect occurrence was extracted from 24,735 documents published between 1999 and 2023. Each record underwent a rigorous validation, wherein the initial extraction was performed by one member of the team, and its suitability was subsequently confirmed by another team member. To ensure accuracy and validity, a third person was involved during the georeferencing phase, conducting a thorough reexamination of the data. The entire team adhered to the same inclusion criteria to maintain consistency and accuracy in incident recordings.

It is essential to ensure precise geographic referencing of the occurrence of diseases. This requires an in-depth review of the original publications and supplementary materials, as well as the analysis of information obtained from different sources. However, due to incomplete location descriptions, it can sometimes be challenging to seamlessly georeference the records. For instance, some articles only mention the HSR mileage coordinates (DK, K) or informal local nomenclature (e.g., pond names in villages)^36–38^. These circumstances necessitated thorough reading of the articles, repeated confirmation using Google and Baidu, and analysis of the semantics obtained from various sources. Therefore, it is necessary to include a “loc_level” field within the dataset to provide readers with insights into the spatial precision associated with each individual record. Lastly, the coordinates extracted using xGeocoding were plotted on Google Earth to ensure that each location was aligned within the correct administrative region of China.

## Usage Notes

Comprehending the geographical distribution of HSR subgrade defects is crucial for hazard prevention and traffic safety management. In response, this study presents the comprehensive compendium on the distribution of HSR subgrade defects in China. The dataset described in this paper is of certain value, as it not only enables the analysis of the spatial and temporal dynamics underpinning defects at various scales, but also proves instrumental in the prediction of safety risk within HSR rail operations. The compilation process of the dataset involves peer-reviewed literature and thorough error-checking, ensuring its reliability and accuracy. The dataset’s architecture is intuitively designed, allowing potential users, such as railway engineering experts, geologists, and policy makers, to easily filter and aggregate the data based on their respective investigation purposes and methodologies.

Our database offers insights for researchers, practitioners, and policymakers in the HSR domain. By consolidating documented defects from academic literature, it provides historical perspectives on subgrade issues. Researchers can identify patterns, explore correlations and assess potential degradation of HSR surfaces, while practitioners and policymakers can use it to inform preventive measures and decision-making for HSR safety and risk management. In addition, we acknowledge the need for a parallel database highlighting effective solutions. Future updates will include a dedicated section to showcase successful case studies and resilient practices.

It is worth noting that the literature considered within this study encompasses China’s “eight vertical and eight horizontal” high-speed railway network, which means that some Class I national railways are also included in the dataset. For instance, railway lines such as the Xiamen-Shenzhen HSR line and the Mudanjiang-Suifenhe HSR line are developed according to different standards compared to the Lanzhou-Xinjiang HSR line and the Harbin-Dalian HSR line. As a result, they may exhibit varying structural resilience to environmental changes.

The dataset utilized in this study is derived from academic literature, representing a compilation of documented defects available in the existing body of knowledge. It is crucial to acknowledge that the dataset may exhibit bias towards railway lines that have been actively studied and published by the rail research community. Therefore, the comprehensiveness of the dataset is contingent upon the extent of academic scrutiny and research coverage within the field. This limitation underscores the need for caution in generalizing the findings to the entire spectrum of high-speed railway infrastructure, as defects on less-studied or undocumented railway lines may not be fully represented. Users should be aware of potential biases in the dataset, as it relies on academic literature that may focus on specific, interesting issues rather than providing a comprehensive distribution of faults across the entire rail network. Interpretation of results should consider this limitation, recognizing the dataset’s emphasis on documented defects from historical case studies within academic literature.

## Data Availability

There is no custom code produced during the collection and validation of this dataset.
